# The impact of collagen membranes on 3D gingival fibroblast toroids

**DOI:** 10.1186/s12903-019-0736-2

**Published:** 2019-03-22

**Authors:** Klara Janjić, Barbara Cvikl, Barbara Schädl, Andreas Moritz, Hermann Agis

**Affiliations:** 1 Department of Conservative Dentistry and Periodontology, University Clinic of Dentistry, Medical University of Vienna, Vienna, Austria; 2 Austrian Cluster for Tissue Regeneration, Vienna, Austria; 3 Ludwig Boltzmann Institute for Experimental and Clinical Traumatology, Vienna, Austria; 4 University Clinic of Dentistry, Medical University of Vienna, Vienna, Austria

**Keywords:** Microtissue, Toroid, Gingiva, Guided tissue regeneration

## Abstract

**Background:**

Development in guided tissue regeneration requires biomaterial testing. 3D cell constructs represent a new approach to bridge the gap between cell culture and animal models. Following the hypothesis that attachment behavior of cells could be observed in toroidal 3D cell constructs, the aim of this study was to evaluate 3D gingival fibroblast (GF) toroids as a simple and feasible in vitro assay to test attachment of oral fibroblasts to collagen membranes.

**Methods:**

3D ring-like structures (toroids) were formed from human GF. Hematoxylin-eosin staining was performed with formed GF toroids. Produced GF toroids were seeded onto plastic surfaces or collagen membranes. The morphology was documented at 24 h, 48 h and 72 h after seeding with light and fluorescence microscopy. Toroid vitality was assessed at same time points with a resazurin-based toxicity assay.

**Results:**

GF showed normal morphology in toroid hematoxylin-eosin staining. Over 72 h, GF toroids on plastic surfaces stayed unchanged, while GF toroids on collagen membranes showed dilatation. GF toroids on plastic surfaces and collagen membranes were metabolically active over the observed period.

**Conclusions:**

Depending on the surface material, 3D GF toroids show different attachment behavior. Thus, GF toroids are suitable as simple assay to study attachment behavior to various biomaterials.

## Background

Applications of guided tissue regeneration (GTR) and guided bone regeneration (GBR) are based on the implementation of a variety of materials to achieve wound healing and finally regeneration in periodontal and bone defects which can occur in consequence of periodontitis [1]. A wide range of biomaterials is in use for GTR approaches and further materials are tested and optimized. A material that is used as a guiding structure for GTR ideally is biocompatible, a barrier between soft and hard tissue, easy to handle and supports regeneration [2]. Collagen membranes are one of the most popular candidates for GTR and have been in clinical use for years [3]. They show potential for GBR applications as for example sinus augmentation [4], providing a basis for implant placement. Additionally, collagen membranes have been shown to be chemoattractive to fibroblasts [5] as well as supportive for attachment of human periodontal fibroblasts [6]. Considering that a collagen membrane should not only function as barrier to enable undisturbed bone healing, but also facilitate regeneration of soft tissue, attachment of fibroblasts is essential [7]. As cell attachment is a pre-requisite for proliferation and migration, it represents a fundamental feature for regeneration. Attention should be paid to the fact that different cell types can have different levels of affinity for different surfaces [8].

To qualify materials for GTR, biomaterial testing starts with traditional 2D cell culture and finally relies on animal experiments and clinical studies. In 2D cell culture, cells are attached to flat plastic wells of cell culture plates, a condition that is not equivalent to that in a tissue and provokes alterations in proliferation, differentiation, apoptosis and gene expression behavior [9]. To close the gap between in vitro and in vivo testing, several approaches for 3D cell cultures are in development where cells form a 3D structure such as spheroids (sphere-like) [9, 10] or toroids (ring-like) [11]. Toroidal microtissues have the advantage to possess a central lumen with a defined diameter which can be considered an artificial wound for in vitro purposes. Toroids can also cluster together and fuse to form larger 3D constructs [12]. Together these studies suggest that microtissues, in particular toroids, might be used as in vitro wound healing assays.

While there is one histological study on spheroids cultured on a collagen barrier membrane for tissue engineering purposes, toroid cell cultures have not been evaluated yet as in vitro bioassays for biomaterials used in oral surgery [10]. Hypothesizing that toroid microtissues could be used as simple assays to observe behavior of tissue on biomaterials, the aim of this study was to test if toroids of human gingival fibroblasts (GF) can be used as simple and feasible in vitro assay to test attachment behavior to collagen membranes.

## Methods

### Cell culture

GF were obtained from tooth donations with oral and written informed consent of the patient at the University Clinic of Dentistry, Medical University of Vienna, Vienna, Austria with approval of the Ethics Committee of the Medical University of Vienna (631/2007). Gingival tissue was removed from uninflamed teeth and cultured in petri dishes containing cell culture medium (α-minimal essential medium; Sigma-Aldrich, St. Louis, MO, USA), supplemented with 10% fetal bovine serum (Gibco, Thermo FischerScientific, MA, USA), penicillin, streptomycin and amphotericin (Gibco, Thermo Fischer). The heterogeneous cell population that grew out from the tissue and consists mainly of fibroblasts was further cultured as GF at 37 °C, 5% CO_2_ and 95% humidity. For experiments, cell passages 4–8 were used. Cells in all cultures and experiments were conducted in α-minimal essential medium with 10% fetal bovine serum and antibiotics and kept at 37 °C, 5% CO_2_ and 95% humidity.

### Toroid culture

**Fig. 1:**
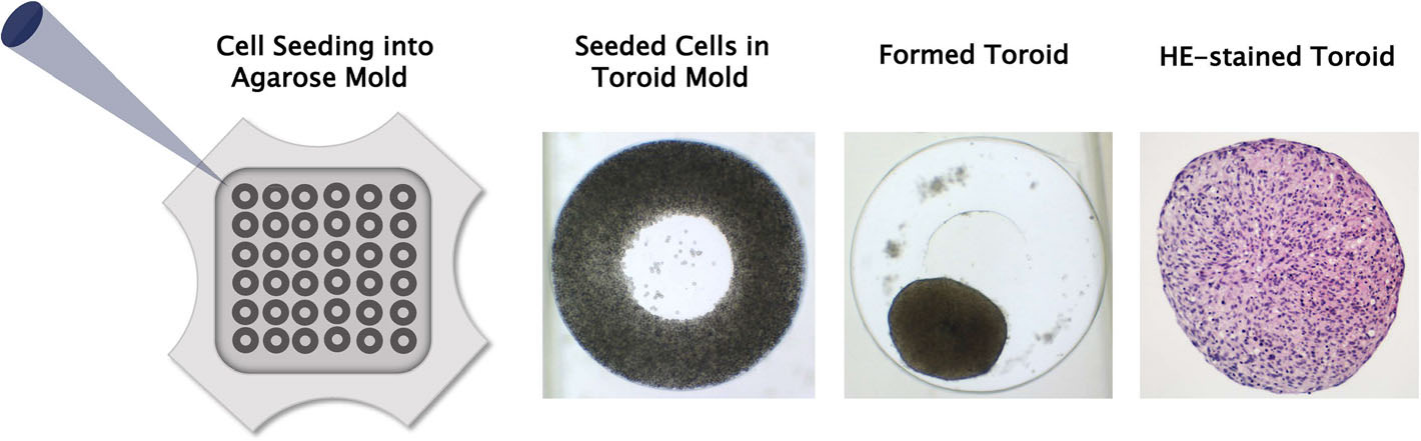
Formation of gingival fibroblast toroids. Human gingival fibroblasts were seeded into agarose molds and evenly settled into toroid-shaped recesses. After 24 h, toroids contracted to a compact 3D structure where the lumen is not visible anymore. HE-stained toroids show numerous basophilic cell nuclei of similar shapes and even distribution

Toroid 3D Petri Dishes® (Microtissues, Inc., Providence, RI, USA) were used to create molds from 2% agarose where one recess for one toroid has following dimensions: 1400 μm outer diameter, 600 μm central diameter, 400 μm width and 750 μm depth (Microtissues, Inc., Providence, RI, USA). A cell suspension of 190 μl containing 1,387,000 GF was pipetted into the molds which were then covered in cell culture medium. After 24 h, GF toroids were harvested and seeded onto plastic surfaces of flat bottom cell culture plates or collagen membranes (Bio-Gide®; Geistlich Biomaterials, Baden Baden, BW, Germany). (Fig. 1) A mean diameter of 445 μm GF toroids (*N* = 4) was measured using ImageJ software (Bethesda, MD, USA).

### Spheroid culture

3D Petri Dishes® (Microtissues, Inc.) for large spheroids were used to create molds from 2% agarose where one recess for one toroid has following dimensions: 800 μm diameter and 800 μm depth (Microtissues, Inc., Providence, RI, USA). A cell suspension of 75 μl containing 547,500 GF was pipetted into the molds which were then covered in cell culture medium. After 24 h, GF spheroids were harvested and seeded onto plastic surfaces of flat bottom cell culture plates or collagen membranes (Bio-Gide®; Geistlich Biomaterials). A mean diameter of 336 μm GF spheroids (*N* = 4) was measured using ImageJ software (Bethesda, MD, USA).

### Monolayer culture

A cell suspension of 100 μl containing 17,000 GF was seeded onto plastic surfaces of flat bottom cell culture plates or collagen membranes (Bio-Gide®; Geistlich Biomaterials) to set up a monolayer culture.

### Attachment score

Attachment of GF toroids, spheroids and monolayers from different donors to plastic or collagen membranes was documented in an attachment score. GF toroids, spheroids and monolayers on collagen membranes underwent careful daily twisting of collagen membranes for microscopy and GF toroids, spheroids and monolayers on plastic and collagen membranes underwent daily medium changes for resazurin-based toxicity assays. For cells that attached 72 h a “+” was noted while a “−” was noted if cells did not attach to plastic or collagen membranes after 72 h, meaning that they fell off the membrane when twisting or were washed away in a medium exchange. Results are displayed in Table 1. For this experiment 5 different donors were used (*N* = 5).

**Table 1:** Attachment score of gingival fibroblast toroids, spheroids and monolayers on plastic or collagen membranes

**Plastic**	**Score**
Toroid	**+ +** − − −
Spheroid	**+ + + **− −
Monolayer	**+ + + + +**
**Collagen Membrane**	**Score**
Toroid	**+ +** − − −
Spheroid	**+ + +** − −
Monolayer	**+ + + + +**

Gingival fibroblast monolayers attached (+) in five of five cases, spheroids in three of five cases and toroids in two of five cases to plastic and collagen membrane surfaces for 72 h, respectively

### Hematoxylin-eosin staining

Hematoxylin-eosin (HE) staining was performed for morphological assessment of GF toroids. Histological assessment was started 48 h after seeding of GF into agarose molds. GF toroids were incubated in Mayer’s hematoxylin for 7 min, following rinsing with distilled water for 30s. Afterwards 0.1% HClOH were added, following a 10 min rinse with tap water and a 30s rinse with distilled water. Subsequently, samples were incubated in 0.5% Eosin G for 3 min, followed by a tap water rinse for 10s. Afterwards, a stepwise dehydration was performed with 70% EtOH for 2 min, 96% EtOH for 2 min, 100% EtOH for 10 min and xylene for 5 min. In the end GF toroids were permanently embedded. Light microscopic images of HE-stained GF toroids were taken in 200-fold magnification.

### Microscopy

**Fig. 2:**
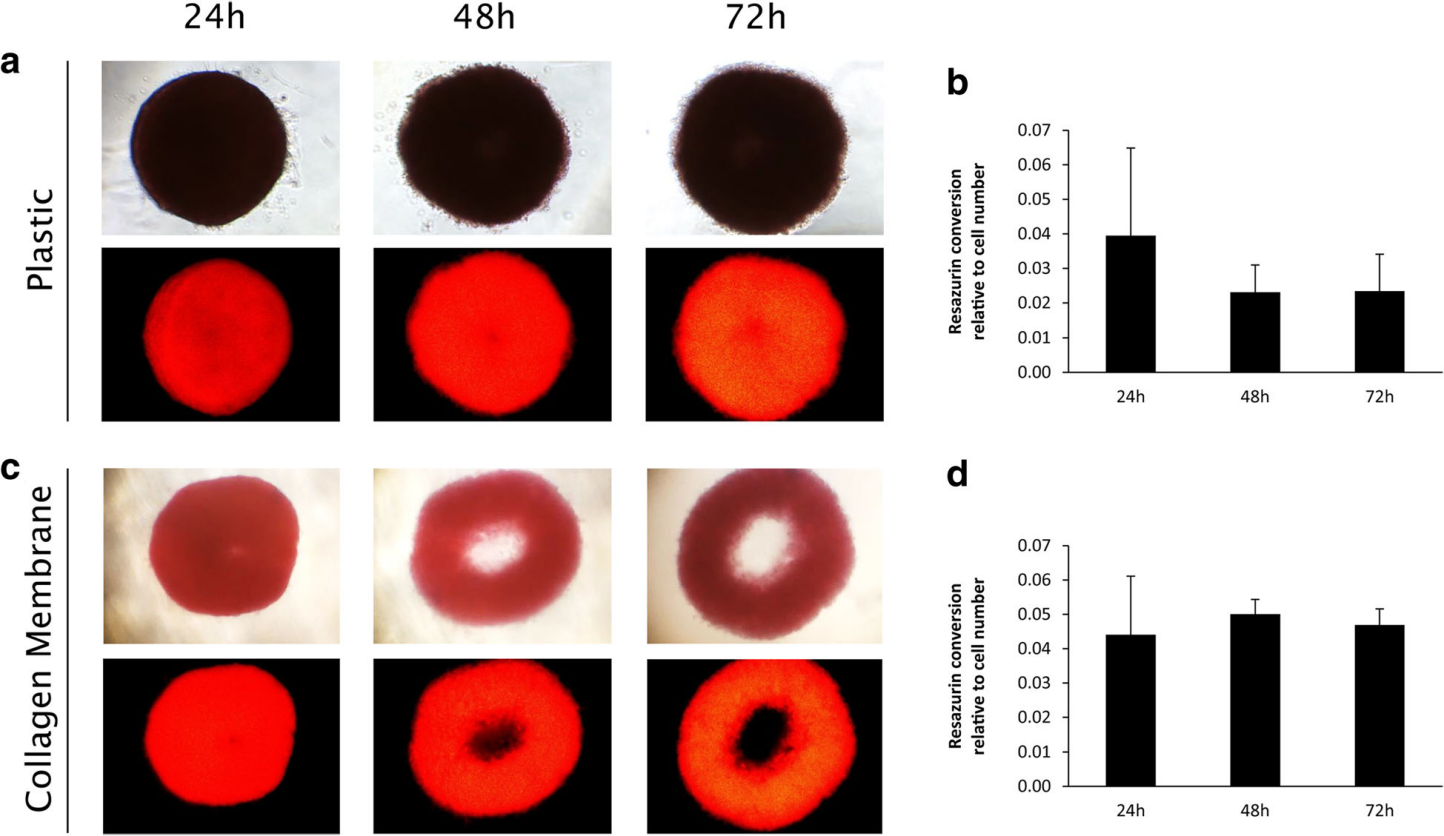
Attachment and metabolic activity of gingival fibroblast toroids on plastic or collagen membranes. Gingival fibroblast toroids on plastic (*N* = 5) (**a**) did not show any changes in shape over 72 h, but were metabolically active as shown in relative fluorescence units (*N* = 3) (**b**). Gingival fibroblast toroids on collagen membranes showed dilatation after 48 h and 72 h (*N* = 5) (**c**) and were metabolically active over the whole observation period (*N* = 3) (**d**). Error bars represent standard deviation

**Fig. 3:**
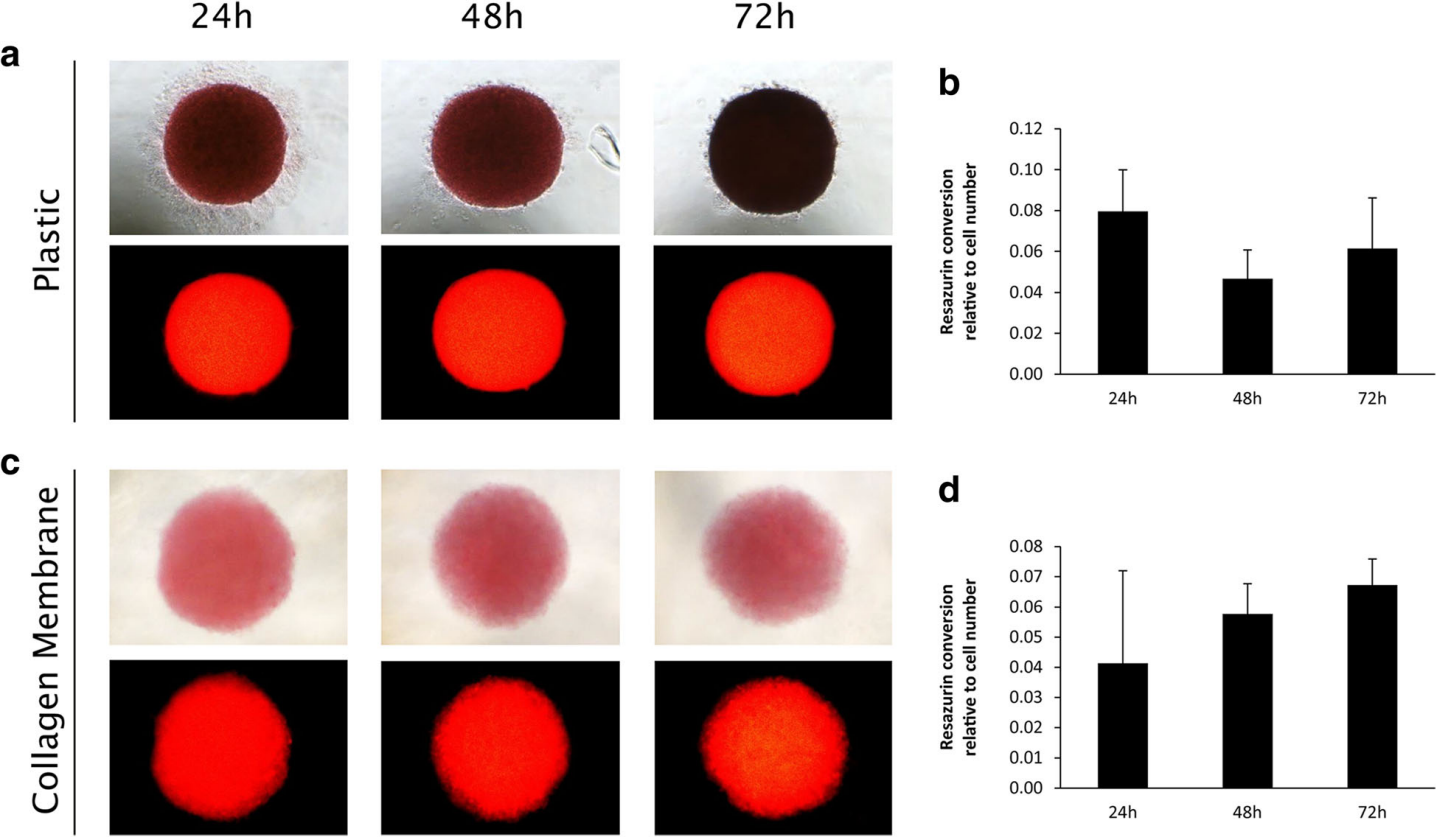
Attachment and metabolic activity of gingival fibroblast spheroids on plastic or collagen membranes. Gingival fibroblast spheroids did not show any changes in shape within 72 h, neither on plastic (*N* = 5) (**a**) nor on collagen membranes (*N* = 5) (**c**). Gingival fibroblast spheroids were metabolically active over the 72 h observation period when seeded on plastic (*N* = 3) (**b**) or collagen membranes (*N* = 3) (**d**), as shown in relative fluorescence units. Error bars represent standard deviation

**Fig. 4:**
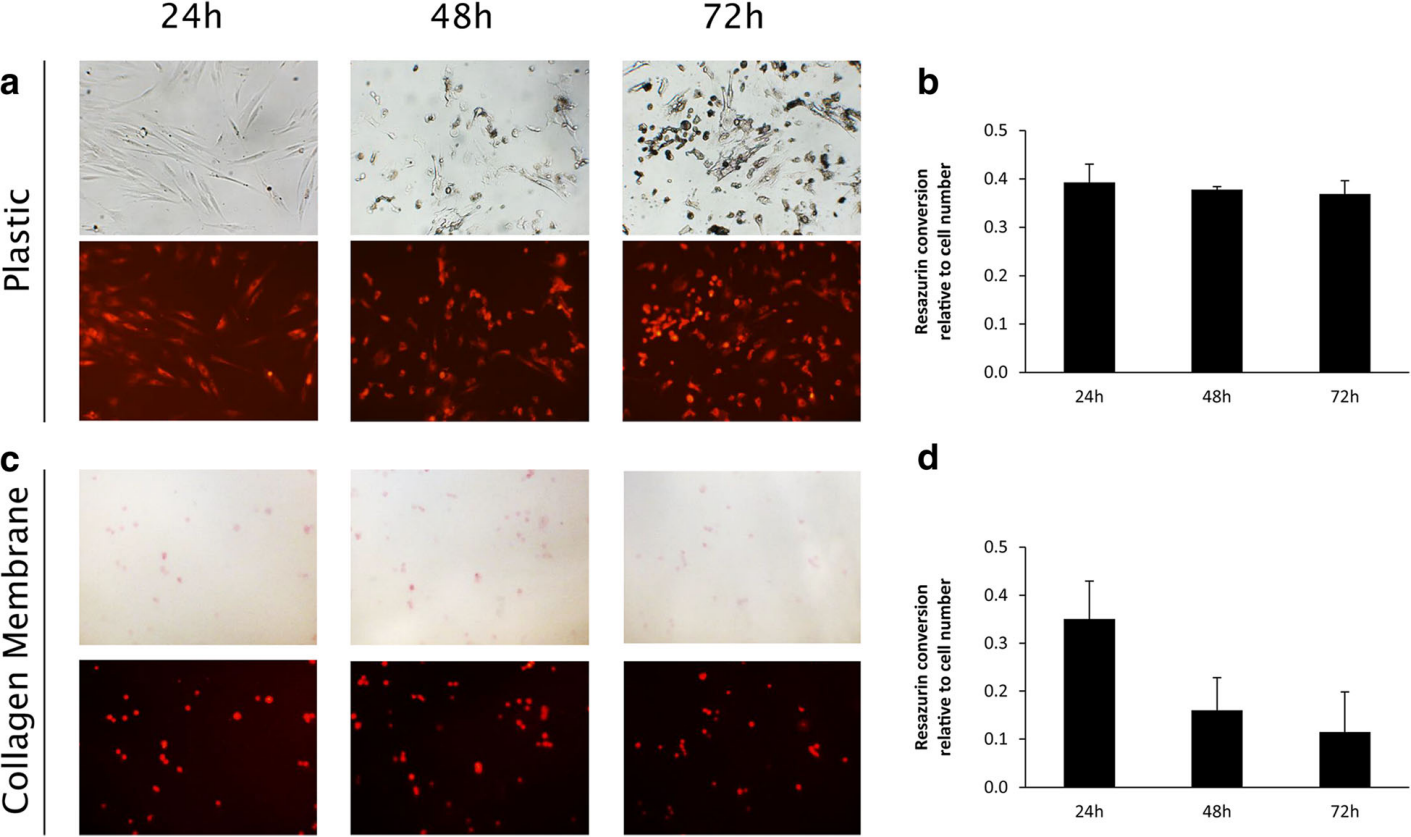
Attachment and metabolic activity of gingival fibroblast monolayers on plastic or collagen membranes. Gingival fibroblast monolayers did not show any changes in shape within 72 h, neither on plastic (*N* = 5) (**a**) nor on collagen membranes (*N* = 5) (**c**). Gingival fibroblast monolayers were metabolically active over the 72 h observation period when seeded on plastic (*N* = 3) (**b**) or collagen membranes (*N* = 3) (**d**), as shown in relative fluorescence units. Error bars represent standard deviation

Light and fluorescence microscopy were used to monitor the attachment process of GF toroids, spheroids and monolayers onto plastic or collagen membranes. Before seeding, GF in suspension were incubated with the fluorescent dye DiI (Thermo Fisher Sctientific, Waltham, MA, USA) for 1 h as described by the manufacturer. The dye is incorporated into the cell membrane and stains the whole cell for tracing GF behavior during the attachment process. Images of GF toroids, spheroids and monolayers on plastic or collagen membranes were taken in 100-fold magnification with a Nikon Diaphot 300 microscope using a brightfield and a green filter for light and fluorescence microscopy, respectively. Images were taken after 24 h, 48 h and 72 h of culture. Microscopy was performed with cells from different donors. Representative images are shown in Figs. 2-4. For this experiment 5 different donors were used (*N* = 5).

### Resazurin-based toxicity assay

The resazurin-based toxicity assay was used to determine cell vitality in terms of metabolic activity in GF toroids, spheroids and monolayers on plastic or collagen membranes. GF were incubated with 10% resazurin solution (Sigma-Aldrich Handels GmbH, Vienna, Vienna, Austria) for 8 h at 37 °C, 5% CO_2_ and 95% humidity. After the incubation period, the supernatant of the cell cultures was transferred to a new plate to proceed with the photometric measurement. Fluorescence of resorufin, produced by metabolizing resazurin, was measured in a Synergy™ HTX Multi-Mode Microplate Reader (BioTek, Winooski, VT, USA) with an excitation wavelength of 540/30 nm and an emission wavelength of 600/40 nm. The assay was performed after 24 h, 48 h and 72 h of culture of GF toroids, spheroids and monolayers on plastic or collagen membranes. Cell culture medium on plastic or collagen membranes without cells was used as blank and subtracted from results of cells on plastic or collagen membranes, respectively. Vitality experiments were performed three times with cells from three different donors in total. For this experiment 3 different donors were used (*N* = 3).

### Statistics

Data of the resazurin-based toxicity assay are displayed as mean + standard deviation, normalized to the total cell number that was seeded initially. The sample size equals 3 (*N* = 3). Data were analyzed with the Kolmogorov-Smirnov Test for normal distribution and significance was evaluated with the Kruskal-Wallis- and the Mann-Whitney-Test. The level of significance was set as *P* < 0.05. All statistical analyses were performed using IBM SPSS Statistics Version 23 (IBM Corporation, Armonk, NY, USA).

## Results

### Gingival fibroblast toroid morphology

Normal morphology was found in HE stainings of GF toroids 48 h after seeding of cells into agarose molds (Fig. 1). Basophilic cell nuclei were distributed over the whole area of the histological slide, sharing similar shapes and sizes.

### Attachment behavior and vitality of gingival fibroblast toroids

The attachment score shows that two out of five GF toroids attached for 72 h to both, plastic and collagen membranes (Table 1).

GF toroids were seeded onto plastic or collagen membranes 24 h after seeding the cells into agarose molds. At this time point, the central lumen of toroids already disappeared (Fig. 1), since cells connect to each other and contract into a dense 3D structure, closing the initial hollow space in the center of the ring. GF toroids stayed in this shape 24 h after seeding them on plastic or collagen membranes. After 48 h and 72 h, GF toroids on plastic still did not show any changes in shape or outgrowth (Fig. 2 a) to the surface while at the same time GF toroids showed a dilatation of the 3D cell construct (Fig. 2 c). There, the initial hollow space in the center re-appears, permitting sight on the underlying collagen membrane.

Both, GF toroids on plastic and on collagen membranes showed metabolic activity in the resazurin-based activity assays. There were no significant changes (*P* > 0.05) in relative fluorescence units between the different time points and surfaces. (Fig. 2 b, d).

### Attachment behavior and vitality of gingival fibroblast spheroids and monolayers

The attachment score shows that three out of five GF spheroids and five out of five GF monolayers attached for 72 h to both, plastic and collagen membranes (Table 1).

No changes of cell arrangement were found in GF spheroids and monolayers on plastic or collagen membranes over the observed time period of 72 h (Fig. 3 a, c & 4 a, c). GF monolayers on plastic show common fibroblast shape (Fig. 4 a), while on collagen membranes cells appear roundish (Fig. 4 c).

GF spheroids and monolayers were metabolically active on plastic and collagen membranes at all time points. No significant differences (*P* > 0.05) were found between the different time points, surfaces and cultures. (Fig. 3 b, d & 4 b, d).

## Discussion

Development of biomaterial testing strategies is of importance in order to improve GTR applications. Testing attachment of soft tissue to new or optimized biomaterials is a substantial aspect for determining qualification of a material for a regenerative therapeutic approach as GTR.

Results of our study show that 3D GF constructs in a toroid shape show different attachment behavior over a determined time frame, depending on the surface material. While on plastic surfaces GF toroids stayed unchanged, on collagen membranes they started to dilate after 48 h hours. This became visible by formation of a central lumen after 48 h which further dilated after 72 h. Further, attachment scores show that GF monolayers attach in each documented case while GF toroids and spheroids do not attach in each case, but fall off from the surface due to twisting of the material or medium exchange. The reason for differences in steadiness of attachment between monolayers and 3D cell constructs has not been published yet.

The formation of 3D cell structures like toroids and spheroids in our study is based on the approach that adherent fibroblasts are not able to attach to agarose, thus they are forced to attach to each other in a shape given by the agarose. Based on this it could be hypothesized that whenever a surface material is not favorable for adherent cells, they prefer to attach to each other [13] as cell-cell interactions in spheroids are intense [14]. This would mean that cells in toroids on plastic move together and keep this formation since it is more efficient than to adhere to the plastic surface while in toroids on collagen membranes cells prefer to attach to the membrane, i.e. spreading out and with that creating a dilating central lumen. It has been reported previously that early adhesion of fibroblast and osteoblast spheroids differs depending on the surface material [14]. In contrast, our study shows that success of early adhesion rather depends on the cell construct, 2D or 3D, than the surface material. Further experiments will be required to clarify underlying molecular mechanisms.

Results of the resazurin-based toxicity assay show that all cell constructs remain vital on plastic and collagen membranes during the time frame of 72 h. For a more precise statement on the metabolic activity, each measured data point should be normalized to the cell number of the respective time point. In our study, results were normalized to the initial cell number of respective culture model. The reason behind this is that currently there is no feasible method available for us to completely separate cells in a 3D form from each other. Further, cells might stay in grooves of the collagen membrane and therefore might not be counted.

In a next step, it would be interesting to evaluate the maximum time span that the cells can stay vital in the spheroid and toroids and to assess if further morphological changes would take place. We have chosen an observation time span of 72 h for this study based on the fact that in GF toroids an effect could be seen after this amount of time, leading to the conclusion that this would be the optimal time frame for our proposed attachment assay by means of feasibility. A limitation of our study is that the observed effect of GF toroids dilating on collagen membranes cannot be evaluated quantitatively. Otherwise a larger sample size would be needed to provide sufficient statistical power. In this study, the sample size was sufficient to demonstrate that the observed effect is reproducible.

Another reason for the observed behavior could be that compared to flat plastic surfaces, collagen membranes offer surfaces with a more relief-like structure. A three-dimensional surface offers more possibility for cells to collect in grooves, spreading their own 3D structure. Relevant surfaces for implant dentistry like titanium or zirconia were already tested for attachment of cells, showing that attachment behavior of periodontal cells differs depending on whether the surface is rough or smooth [15–17].

A previous study showed that cells change in morphology, depending on the pattern of a surface material. There, attachment of periodontal ligament fibroblasts and osteoblast-like cells was tested on different collagen membranes where attached cells showed elongated or round morphology, depending on the membrane type [6]. In our study monolayers of GF attached in an elongated morphology to plastic while they attached in a round morphology to collagen membranes. Previously it was reported that periodontal cells attaching to collagen membranes appear spindle-shaped and flattened in healthy condition. It was proposed that like in 2D monolayer cultures round morphology would suggest rather unhealthy conditions in scanning electron microscopy [18]. In our set up we used an fluorescence microscopy approach which did not allow such in-depth evaluation of the cell morphology on our system. In a previous study it was suggested that Interfiber distance of cells to collagen membranes plays a crucial role in attachment regulation [18]. Periodontal ligament spheroids cultured on collagen membranes or polyglycolic acid membranes showed osteogenic potential when added to dentin [10]. Here, attachment to dentin was increased with spheroids cultured on polyglycolic membranes compared to those cultured on collagen membranes [10], suggesting that attachment onto a specific material one part of a spheroid could influence attachment activity at free sites of the spheroid. To clarify the reasons for attachment preferences and the process of attachment, future studies at molecular levels are required.

## Conclusions

Taken together, our results suggest that attachment behavior of 3D GF toroids varies, depending on the surface material and cell culture model. Thus, GF toroids can only be used as in vitro assays for studying attachment behavior if attachment to the surface is given and stays for at least 72 h. Attachment behavior can easily be observed by the appearance and dilatation of a central lumen or by the absence of central lumen formation in the toroid, respectively. With that 3D GF toroids can be used as a feasible in vitro attachment assay in case of attachment to study cell attachment behavior on various materials.

## Abbreviations

GBR: guided bone regeneration
GF: gingival fibroblasts
GTR: guided tissue regeneration
HE: hematoxylin-eosin
